# Trait Emotional Intelligence and Wellbeing During the Pandemic: The Mediating Role of Meaning-Centered Coping

**DOI:** 10.3389/fpsyg.2021.648401

**Published:** 2021-05-13

**Authors:** Maria-Jose Sanchez-Ruiz, Natalie Tadros, Tatiana Khalaf, Veronica Ego, Nikolett Eisenbeck, David F. Carreno, Elma Nassar

**Affiliations:** ^1^Department of Psychology, Lebanese American University, Byblos, Lebanon; ^2^Department of Psychology, University of Almería, Almería, Spain; ^3^Graduate Studies and Research Office, Lebanese American University, Byblos, Lebanon

**Keywords:** existential positive psychology, trait emotional intelligence, coping, meaning-centered coping, wellbeing

## Abstract

Studies investigating the COVID-19 pandemic from a psychological point of view have mostly focused on psychological distress. This study adopts the framework of existential positive psychology, a second wave of positive psychology that emphasizes the importance of effective coping with the negative aspects of living in order to achieve greater wellbeing. Trait emotional intelligence (trait EI) can be crucial in this context as it refers to emotion-related personality dispositions concerning the understanding and regulation of one’s emotions and those of others. The present study investigated the relationship between trait EI and both wellbeing and psychological distress (i.e., depression, anxiety, and stress), while exploring the mediating role of meaning-centered coping (proactive transformative strategies based on meaning in life) and maladaptive coping (i.e., behavioral disengagement and self-blame) during the first few months of the COVID-19 pandemic. A sample of 326 Lebanese adults completed measures of trait EI, wellbeing, psychological distress, coping, and meaning-centered coping. Results showed a strong positive correlation between trait EI and meaning-centered coping. Trait EI also correlated positively with wellbeing and negatively with psychological distress. Structural equation modeling showed that meaning-centered coping partially mediated the relationship between trait EI and wellbeing. Maladaptive coping fully mediated the relationship between trait EI and psychological distress. Findings indicate that trait EI is positively related to dealing with a stressful situation such as the pandemic in positive ways at both the cognitive level, by reformulating the situation to see something valuable in it, and behavioral level, by being proactive about it. Trait EI was positively linked to seeing the situation as an opportunity for personal growth, finding personal meaning in this situation, maintaining an attitude of hope and courage, and acting more responsibly with one’s self and others during the current crisis. In turn, this coping formula was related to lower psychological distress and improved mental health. These results are consistent with the existential positive psychology framework and can inform implementation programs and policies aiming at raising awareness and promoting healthy and successful coping during the pandemic.

## Introduction

An outbreak of coronavirus disease (COVID-19) emerged in China by the end of 2019 and was quickly declared a global pandemic (World Health Organization, 2020a). The first case in Lebanon was identified on February 21, 2020 (Knecht and Francis, 2020), with the number of cases steadily increasing throughout the following months. Several sources, including the Lebanese Ministry of Public Health, began advising the public to follow the safety measures, including hygiene-related practices and social distancing. Lebanese were also advised to take care of their mental health and wellbeing, especially when it comes to their stress and anxiety (World Health Organization, 2020b).

Research on this outbreak in relation to psychological health is still scarce, with the majority of the available studies having mainly investigated the high levels of anxiety, depression, and stress in different populations due to the pandemic, including university students (Wang et al., 2020) and international samples with a wider age range (Ozamiz-Etxebarria et al., 2020; Rodríguez-Rey et al., 2020; Sood, 2020). In Lebanon, a recent study exploring the impact of the pandemic on mental health found that increased fear of the virus was associated with higher levels of anxiety and stress (Salameh et al., 2020), and another study reported that more than half the sample studied (717 youth) were experiencing moderate to high levels of anxiety and depression during the pandemic (Sanchez-Ruiz et al., forthcoming).

In such an adverse scenario, a new paradigm in psychology called *existential positive psychology* (Wong, 2011) can be particularly valuable. Existential positive psychology, considered by some to be the second wave of positive psychology, combines positive psychology with existential humanistic components (Wong, 2019). According to Wong (2011), this perspective shifts from mainstream clinical psychology, which places primary emphasis on the reduction of psychological distress, to an approach that emphasizes the acceptance of both suffering and joy as parts of life that are necessary for growth. This paradigm represents an advancement of the first wave of positive psychology (Seligman and Csikszentmihalyi, 2000), which presented a revolutionary perspective on psychological wellbeing and has been criticized for being excessively focused on positivity, while overlooking negative emotions and events or considering them as the absence of positive aspects (Held, 2004; Wong and Roy, 2018). However, according to the existential positive psychology framework, crises such as the current pandemic, although generally undesirable, can also serve as promoters of personal development. This dialectical way of coping with the present crisis—by approaching both its negative and positive aspects—can be crucial in this regard.

While the majority of psychological research on this outbreak has focused on negative outcomes such as anxiety, depression, and stress, the novelty of this study is the investigation of effective psychological coping with the pandemic under the framework of existential positive psychology. From this approach, the pandemic is viewed as an opportunity for growth—a challenge rather than a threat—and suggests that effective coping might have numerous positive psychological outcomes, especially when it comes to our overall happiness and wellbeing. One of the most extended approaches to happiness and wellbeing is the PERMA model (Seligman, 2011). Seligman (2011) defined wellbeing as being composed of positive emotion, engagement, relationships, meaning in life, and accomplishment (PERMA). Indeed, the mentioned factors have been strongly correlated with flourishing and life satisfaction (Ryff, 2013; Butler and Kern, 2016; Ryff et al., 2016). However, how these five factors of wellbeing can be sustained during the current global crisis is still being investigated. In this study, we explored the role of two important factors that may be particularly important in the maintenance of wellbeing during the COVID-19 pandemic, namely, trait emotional intelligence (EI) and meaning-centered coping.

### Trait Emotional Intelligence and Wellbeing During the Pandemic

Personality and emotion-related predispositions prone individuals to cope with external situations in different ways, which in turn results in a myriad of behaviors, psychological states, and life outcomes. In the same line, previous literature has shown the association between trait EI and different coping strategies that foster wellbeing and reduce psychological distress (Davis, 2018; Keefer et al., 2018). Trait emotional intelligence (EI), which refers to emotion-related self-perceptions and dispositions, is a determinant personality factor that can significantly contribute to effective coping in stressful situations. Trait EI is defined by Petrides et al. (2018) as “how good we believe we are in terms of understanding, managing, and utilizing our own and other people’s emotions.” This conceptualization of trait EI is distinct from the construct of ability EI (Brannick et al., 2009). While trait EI refers to a “constellation of self-perceptions located at the lower levels of personality hierarchies” (Petrides et al., 2007) and is assessed via self-report, ability EI is concerned with one’s ability to engage in emotion-related cognitive abilities (Salovey and Mayer, 1990), and it is best measured through maximum-performance tests (see Siegling et al., 2015 for more details on this distinction).

Studies have consistently shown trait EI, or certain factors within the construct (see Table 1 for the trait EI sampling domain), to be a significant predictor of several positive life outcomes (see Petrides et al., 2016 for a review). For instance, individuals with higher scores on trait EI have shown lower psychological and physiological reactivity to stress (Mikolajczak et al., 2007) and lower levels of depression and physical pain (Mavroveli et al., 2007) compared with their low trait EI counterparts. Systematic reviews and meta-analyses show that trait EI is linked to good overall health, including lower levels of depression, anxiety, and distress in particular (Martins et al., 2010; Lea et al., 2019) along with a low risk for both suicidal ideation and attempts (Domínguez-García and Fernández-Berrocal, 2018). Trait EI also moderated negative mood changes after an experimental stressor (Mikolajczak et al., 2009), and the Self-control and Emotionality trait EI factors significantly predicted aggressive behavior (Sanchez-Ruiz and Baaklini, 2018).

**TABLE 1:** The sampling domain of trait emotional intelligence (EI) in adults.

Global trait EI	High scorers perceive themselves as…
***Wellbeing***	
Self-esteem	…successful and self-confident.
Trait happiness	…cheerful and satisfied with their lives.
Trait optimism	…confident and likely to “look on the bright side” of life.
***Self-Control***	
Emotion regulation	…capable of controlling their emotions.
Stress management	…capable of withstanding pressure and regulating stress.
Impulse control	…reflective and less likely to give into their urges.
***Emotionality***	
Emotion perception (self and others)	…clear about their own and other people’s feelings.
Emotion expression	…capable of communicating their feelings to others.
Relationships	…capable of having fulfilling personal relationships.
Trait empathy	…capable of taking someone else’s perspective
***Sociability***	
Social awareness	…accomplished networkers with excellent social skills.
Emotion management (others)	…capable of influencing other people’s feelings.
Assertiveness	…forthright, frank, and willing to stand up for their rights.
Adaptability*	…flexible and willing to adapt to new conditions.
Self-motivation*	…driven and unlikely to give up in the face of adversity.

*These two facets feed directly into the global trait EI score without going through any factor. Adapted from “Developments in Trait Emotional Intelligence Research,” by K.V. Petrides et al., 2016, Emotion Review, 8(4), p. 336 (https://doi.org/10.1177/1754073916650493). Copyright 2016 by the American Psychological Association.

Trait EI has a crucial role within positive psychology, which emphasizes human performance, adjustment, positive emotions, and character strengths (e.g., Allen et al., 2014). In fact, trait EI has repeatedly been associated with wellbeing (Por et al., 2011; Bhullar et al., 2012), optimism (Bartz et al., 2018), academic achievement (Sanchez-Ruiz et al., 2013, 2019), goal setting (Spence et al., 2004), prosocial behavior (Mavroveli and Sánchez-Ruiz, 2011), life satisfaction (Austin et al., 2005; Liu et al., 2013), and character strengths (Ros-Morente et al., 2018). A meta-analysis by Sánchez-Álvarez et al. (2016) confirmed that high scores on EI were related to wellbeing, along with other positive outcomes such as resourcefulness, meaningfulness, and positive affectivity. Additionally, Furnham and Petrides (2003) found that trait EI accounted for more than 50% of variance in happiness over and above the Big Five personality dimensions. It has been recommended for future research on trait EI to focus on potential mediators between trait EI and health and wellbeing (Zeidner et al., 2012). The present study aims to contribute in this direction by examining the relationship between trait EI and wellbeing through different coping mechanisms, and in particular meaning-centered coping.

### Trait Emotional Intelligence, Coping Strategies, and Meaning-Centered Coping

Another important factor to be considered during this pandemic because of its influence on wellbeing is coping. According to Pearlin and Schooler (1978), “coping refers to behavior that protects individuals from being psychologically harmed by problematic social experience.” There are different models of coping in the literature, such as avoidance- and approach-based coping [e.g., Eisenberg et al. (2012)], problem- and emotion-focused coping (e.g., Lazarus and Folkman, 1984), and that by Zeidner and Saklofske (1996), who categorized coping strategies as adaptive or maladaptive. According to these authors, adaptive coping strategies are described as those that “effectively eliminate the stressor or reduce its negative emotional impact,” whereas maladaptive ones are those that either do not change the situation or make it worse, thus “leading to prolonged exposure to stress and elevated levels of anxiety and other negative emotions” (see Keefer et al., 2018).

To date, there is no consensus in the literature that favors a particular model over others. In addition, criticisms revolving around the various models are important to consider. For instance, Carver et al. (1989) showed that certain coping strategies, such as seeking emotional support, can be considered as both a problem-focused and an emotion-focused coping strategy, and then subsequently be adaptive and/or maladaptive depending on situational factors and the way the coping strategy is used. In the present study, we have adopted the adaptive vs. maladaptive model.

Several studies have analyzed the relationship between trait EI and different types of coping. A study by Enns et al. (2018) found that trait EI correlated negatively with perceived stress, and adaptive coping was found to mediate that relationship. In addition, Gawali (2012) conducted studies on the relationship between trait EI and coping among teachers, finding that trait EI predicted positive and adaptive coping. Many other studies have confirmed the association between trait EI and the use of adaptive coping strategies, in addition to adequate access to psychosocial resources, such as social support (Campbell and Ntobedzi, 2007; Mavroveli et al., 2007; Zeidner et al., 2012). Trait EI has also been associated with active coping and planning (Enns et al., 2018) and instrumental support (Gawali, 2012), which are key components of transformational coping (Omeri et al., 2004), a coping style that is akin to meaning-centered coping.

Given the existential impact of COVID-19 on individuals’ lives, a coping style directly aimed to sustain meaning in life may be of clinical relevance. Meaning in life is defined as the “cognizance of order, coherence, and purpose in one’s existence, the pursuit and attainment of worthwhile goals, and an accompanying sense of fulfillment” (Reker and Wong, 1988). This construct has been associated with the enhancement of wellbeing along with decreased distress (Vos and Vitali, 2018) and other negative psychological outcomes, especially depression (Carreno et al., 2020) and even suicide (Lew et al., 2019). Based on the abovementioned considerations, a coping style that focuses on re-creating meaning and purpose in life can be crucial to maintain wellbeing through the pandemic (Eisenbeck et al., unpublished data). Meaning-centered coping in this context is understood as a set of attitudinal and behavioral strategies including the maintenance of hope and courage, positive reframing, life appreciation, and engagement in meaningful prosocial activities (Eisenbeck et al., unpublished data). This coping style is rooted in Frankl’s (1969, 1984) approach to meaning and its posterior development by Wong under the existential positive paradigm (Wong, 1993, 2020; Wong et al., 2006). Rather than identifying with the outer circumstances and entering in a circle of suffering, transcending them and accepting the pleasures and displeasures in life can restore the balance needed to face stressors in life (e.g., Wong, 1993).

High trait EI scorers tend to perceive stressors as a challenge rather than a threat (Mikolajczak and Luminet, 2008), which facilitates access to psychosocial resources (Frederickson et al., 2012; Keefer et al., 2018), and such challenges can make them more inclined to find meaning and purpose in life under stressful circumstances. Trait EI might indeed have a close relationship with meaning-centered coping. Many of the notions encompassed by this coping style such as acceptance, the maintenance of hope, and the use of courage are emotional and psychologically mature in essence, and thus require the strong foundations of emotional perception, understanding and regulation, which are part of trait EI’s sampling domain. However, no previous study has explored the relationship between these two constructs empirically.

### The Present Study

In Lebanon, only a few studies exploring the relationships between trait EI and other variables have been conducted, and these have shown that trait EI relates to academic achievement (Sanchez-Ruiz and El Khoury, 2019), moderates social media use (Zeeni et al., 2018), and protects against the deleterious effects of body image dissatisfaction (Sanchez-Ruiz et al., 2020). However, to our knowledge, no studies have examined the role of trait EI in the context of the pandemic in Lebanon. Internationally, we have identified only one mixed-methods study that took place in Poland and found that trait EI played a protective role in reducing the intensity but not the frequency of experiencing negative emotions during the pandemic (Moroń and Biolik-Moroń, 2021).

In addition to exploring this topic for the first time in an under-researched context, this study investigates the influence of trait EI in adapting to critical situations in a novel way, analyzing the role of trait EI in wellbeing during the unprecedented pandemic from the lens of the existential positive psychology framework through adaptive and meaning-centered coping. That is, people with high trait EI may adjust better to adversity because they are more likely to adopt a meaning-centered coping style.

Based on the previously reviewed literature, the present study aims to test the following hypotheses:

(H1) Trait EI will negatively correlate with psychological distress (i.e., depression, anxiety, and stress), and maladaptive coping, and positively with wellbeing, adaptive coping, and meaning-centered coping.
(H2) The relationship between trait EI and psychological distress (i.e., depression, anxiety, and stress) will be mediated by maladaptive coping.
(H3) The relationship between trait EI and wellbeing will be mediated by adaptive coping and meaning-centered coping.

## Materials and Methods

### Participants

The sample consisted of 360 Lebanese individuals (210 females) aged between 18 and 69 years old (*M* = 29.55, *SD* = 12.37) who filled out an online survey between April 27 and June 6, 2020. The participants were a diverse national sample from different areas in Lebanon. Participants diagnosed with any psychological disorder were excluded from the analysis. More details on the distribution of the different socio-economic variables are presented in Table 2. About half of the participants were single, and very few were widowed or divorced. Moreover, most participants were educated with at least a Bachelor’s degree and about 70% categorized their economic status as average.

**TABLE 2:** Distribution of the demographic and socioeconomic variables.

Variables		*n* (%)
Gender	Female	210 (64.4)
	Male	116 (35.6)
Social Status	Single	174 (53.4)
	In a relationship	70 (21.5)
	Married	72 (22.1)
	Widowed	2 (0.6)
	Divorced	8 (2.4)
Economic Status	Above average	64 (19.6)
	Average	240 (73.6)
	Below average	22 (6.7)
Educational Level	Elementary or lower	1 (0.3)
	High School	23 (7.0)
	Bachelor or Master	163 (50)
	Ph.D.	38 (11.7)
	Still a student	101 (31.0)
Religiosity	Part of a religious organization	91 (27.9)
	Religious on their own way	153 (46.9)
	Not religious	73 (22.4)
	Not sure	9 (2.8)

### Measures

1. The Depression, Anxiety, and Stress Scale (DASS-21; Lovibond and Lovibond, 1995) is a 21-item scale that consists of three subscales, namely, depression, anxiety, and stress (e.g., “I tended to over-react to situations”). The total score indicates general psychological distress. Participants are asked to rate the frequency of certain negative emotional experiences during the past week on a four-point Likert scale ranging from 0 (not applicable to me at all) to 3 (very applicable to me). The internal consistencies of the DASS-21’s scores in this study were as follows: Total = 0.93, depression = 0.90, anxiety = 0.81, and stress = 0.86.
2. The PERMA Profiler (Butler and Kern, 2016) is a measure of subjective wellbeing and satisfaction. It is a 23-item scale that consists of five subscales: Positive emotions, engagement, relationships, meaning in life, and accomplishment (e.g., “How often do you achieve the important goals you have set for yourself?”). A seven-point Likert scale ranging from 0 to 6 was used instead of the usual 10-point Likert scale to be consistent with the remaining survey questions. The internal consistencies of the scale’s scores in this sample were as follows: Total = 0.93, positive emotions = 0.87, engagement = 0.62, relationships = 0.79, meaning in life = 0.90, and accomplishment = 0.78.
3. The Brief Coping Orientation to Problems Experienced (Brief COPE; Carver, 1997) is a 28-item scale organized into 14 subscales: Active coping, planning, instrumental support, use of emotional support, self-distraction, relief, positive reinterpretation, denial, acceptance, religion, substance use, humor, self-blame, and behavioral disengagement (e.g., “I’ve been giving up attempting to deal with it”). It is rated on a four-point Likert scale ranging from 0 (I never do this) to 3 (I always do this). The internal consistencies of the subscales’ scores ranged from 0.57 to 0.87, except for self-distraction, which had a low Cronbach’s alpha of 0.39 and was thus eliminated from the analyses.
4. The Meaning-Centered Coping Scale (MCCS; Eisenbeck et al., unpublished data) was developed as part of a broader research project to gather information about participants’ coping strategies with the current pandemic from the existential positive psychology perspective. It consists of nine items and measures maintenance of hope and courage, positive reframing, life appreciation, and engagement in meaningful prosocial activities. It is rated on a seven-point Likert scale ranging from 1 (not at all agree) to 7 (completely agree), and sample items include “I have found a personal meaning in the current situation” and “I help others during this time.” The internal consistency of the total scores in this sample was 0.89.
5. The Trait Emotional Intelligence Questionnaire (TEIQue-SF; Petrides, 2009) is a 30-item measure of trait EI organized into 15 facets and four factors: Wellbeing, emotionality, self-control, and sociability (e.g., “I’m usually able to influence the way other people feel”). A global (total) trait EI score is also reported, and items are rated on a seven-point Likert scale ranging from 1 (completely disagree) to 7 (completely agree). The full form, English version of the TEIQue (v.1.50) has been previously validated in Lebanon and has shown excellent psychometric properties (Sanchez-Ruiz et al., 2021). The short-form, English version of the TEIQue has not been validated in Lebanon to date; however, several studies have reported excellent reliability scores of the TEIQue-SF in the country (see Sanchez-Ruiz and Baaklini, 2018; Gillespie-Lynch et al., 2019; Sanchez-Ruiz et al., 2019). The internal consistencies of the scale’s scores in this sample were as follows: Global trait EI = 0.90, wellbeing = 0.84, self-control = 0.68, emotionality = 0.65, and sociability = 0.70.

### Procedure

Invitations to fill out the online survey were sent to participants via email and text messages after obtaining ethical approval from the Institutional Review Board. The informed consent and questionnaire were designed online and took around 20 min to complete. Participation in the study was completely voluntary. The inclusion criteria were being over the age of 18 and a Lebanese national residing in Lebanon.

### Statistical Analyses

First, we ran bivariate correlations between study variables to identify the coping strategies that were mostly related to wellbeing and psychological distress. Variables included in the structural model were selected based on these criteria. Bonferroni’s correction was applied to reduce Type I error rate. Next, a measurement model was tested using confirmatory factor analysis including the chosen coping variables. Regression paths were then added to construct the final structural model, which showed satisfactory fit indices. The maximum likelihood estimation method with the Satorra–Bentler correction (MLM) was used for the structural equation model since the distribution of the data showed to be deviating from multivariate normality. Robust estimates of the root-mean-squared-error-of-approximation (RMSEA), Tucker–Lewis index (TLI), and chi-square test statistic are provided to assess the model fit as suggested by Savalei (2018) in the case of non-normal data. An RMSEA value that is less than 0.06 is considered good and acceptable if less than 0.08 (Lei and Wu, 2007; VanderWeele and Vansteelandt, 2010). A value of the TLI greater than 0.9 is considered to be good and excellent when greater than 0.95 (Hu and Bentler, 1998; VanderWeele, 2012). Ideally, the chi-square statistic should be non-significant when the model presents a good fit. However, it is usually not a strict condition for judging model fitness due to its sensitivity to sample size (Hu and Bentler, 1998). All statistical analyses were conducted using R Version 4.0.3.

## Results

### Variable Selection and Assessment of the Measurement Model

A data-driven approach was used in this study to categorize the coping subscales as “adaptive” or “maladaptive” in order to address the previously mentioned disparity in the literature regarding different models of coping and related criticisms. Accordingly, coping strategies were considered “adaptive” if they had significant negative correlations with all of the psychological distress variables (i.e., anxiety, depression, and stress) and a significant positive correlation with wellbeing. On the other hand, coping strategies that correlated positively with all the psychological distress variables and negatively with wellbeing were considered to be “maladaptive.” This is in line with the conceptualizations of adaptive and maladaptive coping strategies as explained by Zeidner and Saklofske (1996).

Correlations among study variables are presented in Table 3. Results showed that wellbeing, as measured by the PERMA profiler, and the psychological distress indicators stress, anxiety, and depression, as measured by the DASS-21, were significantly correlated to behavioral disengagement (*r* = −0.40, *r* = 0.44, *r* = 0.50, *r* = 0.53, respectively), self-blame (*r* = −0.41, *r* = 0.48, *r* = 0.50, *r* = 0.55, respectively), and meaning-centered coping (*r* = 0.65, *r* = −0.30, *r* = −0.24, *r* = −0.51, respectively). Moreover, trait EI was found to positively correlate with active coping (*r* = 0.34) and positive reframing (*r* = 0.33), as well as to negatively correlate with behavioral disengagement (*r* = −0.48) and self-blame (*r* = −0.45). All reported correlations were significant to the 0.05 level after the application of Bonferroni’s correction.

**TABLE 3:** Correlations among study variables.

	Wellbeing	Anxiety	Depression	Stress	Trait EI
**Brief-COPE**					
Active Coping	**0.34**	−0.10	**−0.30**	**−0.19**	**0.34**
Denial	−0.12	0.16	0.08	0.12	−0.13
Substance Use	−0.11	0.12	0.11	0.10	−0.09
Emotional Support	**0.20**	0.11	0.00	0.14	0.05
Instrumental Support	0.11	0.13	−0.02	0.13	0.04
Behavioral Disengagement	**−0.40**	**0.50**	**0.53**	**0.44**	**−0.48**
Venting	−0.01	**0.27**	0.18	**0.32**	−0.02
Positive Reframing	**0.24**	−0.05	−0.17	−0.07	**0.33**
Planning	0.17	0.10	−0.06	0.04	0.17
Humor	−0.03	0.09	0.10	0.07	0.06
Acceptance	0.17	−0.10	−0.09	−0.08	0.17
Religion	0.05	0.10	−0.10	0.01	0.14
Self-Blame	**−0.41**	**0.50**	**0.55**	**0.48**	**−0.45**
Meaning-Centered Coping	**0.65**	**−0.24**	**−0.51**	**−0.30**	**0.60**
Trait EI	**0.75**	**−0.44**	**−0.66**	**−0.52**	**1.00**
Trait Wellbeing	**0.77**	**−0.37**	**−0.64**	**−0.44**	**0.86**
Trait Sociability	**0.51**	**−0.29**	**−0.45**	**−0.34**	**0.75**
Trait Emotionality	**0.51**	**−0.27**	**−0.43**	**−0.29**	**0.74**
Trait Self Control	**0.54**	**−0.47**	**−0.54**	**−0.55**	**0.80**

*Correlations in bold are significant after application of the Bonferroni correction at the 5% level.*

A confirmatory factor analysis was conducted to test the measurement model with trait EI factors loading on one latent variable, and behavioral disengagement and self-blame loading on another. The latter was labeled as maladaptive coping due to its significant positive correlation with psychological distress. The measurement model showed excellent fit, with all loadings being higher than 0.50 (RMSEA = 0.049; TLI = 0.979; χ^2^ = 12.155, df = 8, *p* = 0.144).

### The Structural Equation Model

**FIGURE 1:**
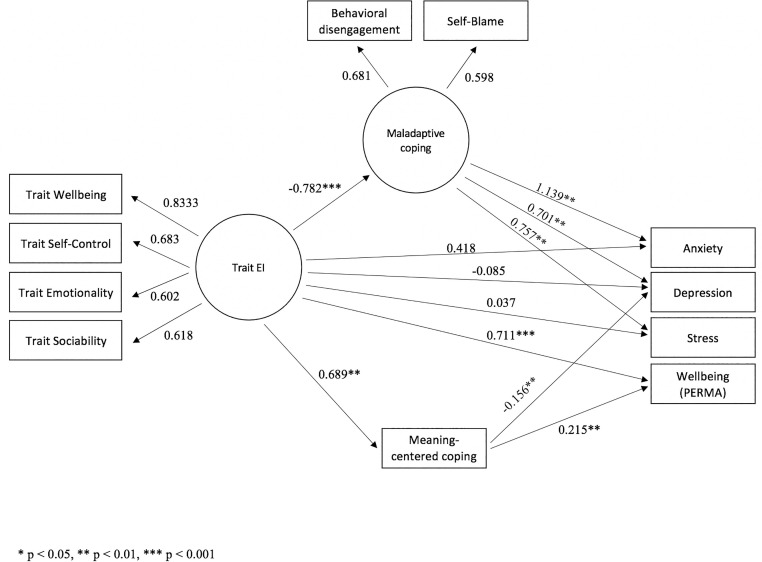
Structural equation model predicting anxiety, depression, stress, and wellbeing (PERMA).

The measurement model was integrated in a full structural equation model to study the suggested hypotheses. The standardized path weights are shown in Figure 1 on the final structural model, which showed satisfactory fit indices (RMSEA = 0.067; TLI = 0.959; χ^2^ = 61.754, df = 32, *p* = 0.001). The relationship between maladaptive coping and anxiety might seem anomalous due to a standardized regression weight greater than 1. However, the appearance of such coefficients is perfectly legitimate and is probably due to high correlation between maladaptive coping and trait EI (Deegan, 1978). When removing the non-significant path, due to mediation, between trait EI and anxiety, the weight between maladaptive coping and anxiety becomes 0.741 and remains significant.

We investigated the potential overlap between the wellbeing factor of trait EI and wellbeing as measured by PERMA. From a theoretical point of view, the constructs are related but differ in that the wellbeing factor of trait EI measures personality predispositions toward being optimistic and having a positive outlook about life and oneself, while PERMA is a broad measure of frequency of behaviors, thoughts, and feelings that indicate life satisfaction (e.g., regarding accomplishment, goal seeking, flow experiences, sense of direction, etc.). In order to rule out colinearity between predictor and outcome, we removed trait EI wellbeing from the model, and the relationship between trait EI and wellbeing (PERMA) remained significant with a weight of 0.593.

Our model reveals that maladaptive coping fully mediates the relationship between trait EI and psychological distress, while meaning-centered coping partially mediates the relationship between trait EI and wellbeing. Table 4 shows the direct and indirect effects of the mediators tested for the different relationships.

**TABLE 4:** Direct and indirect effects from mediation testing.

		Standardized parameter	*p*-value
Maladaptive coping mediating the relationship between trait EI and depression	Direct	−0.085	0.687
	Indirect	−0.548	0.005
Maladaptive coping mediating the relationship between trait EI and anxiety	Direct	0.418	0.164
	Indirect	−0.890	0.003
Maladaptive coping mediating the relationship between trait EI and stress	Direct	0.037	0.870
	Indirect	−0.592	0.008
Meaning-centered coping mediating the relationship between trait EI and wellbeing	Direct	0.711	<0.001
	Indirect	0.148	<0.001

## Discussion

The findings of the present study show support for the hypothesized relationships between trait EI and depression, anxiety, stress, and wellbeing (H1). These findings are consistent with previous literature, including the meta-analyses by Martins et al. (2010) and Schutte et al. (2007) who found that trait EI predicted lower psychological distress and burnout, as well as higher mental health. This is also expected due to the positive influence of trait EI in virtually every life domain, including psychological adjustment (Petrides et al., 2016). These findings are particularly important in the context of the pandemic, as trait EI showed to maintain its negative relationship with these indicators of psychological distress.

In addition, trait EI was associated with maladaptive coping, wellbeing, and meaning-centered coping, whereby all the aforementioned correlations had strong to moderate effect sizes. “Adaptive coping” could not be incorporated as a separate variable in our analysis because none of the coping strategies had significant negative correlations with all three psychological distress indicators and a significant positive correlation with wellbeing. As a result, the hypothesis on the positive relationship between trait EI and adaptive coping was not fully met. Nevertheless, significant correlations were found between trait EI and the active coping and positive reframing strategies in particular, both of which partially met our criteria for “adaptive coping.” The negative relationship between trait EI and maladaptive coping (behavioral disengagement and self-blame) and positive relationship with active and positive reframing coping are also in line with the literature and have been replicated across various populations (Horwitz et al., 2011; Keefer et al., 2018). Prentice et al. (2020) showed that EI, as measured by Law et al. (2004)’s self-report EI scale (WEIS), has been linked to enhanced task-oriented and emotion-oriented coping with the pandemic. However, future studies will need to replicate these findings using a trait EI measure. Additionally, higher trait EI scores were associated with less frequent anger, disgust, and sadness, and more frequent positive states, including happiness, over the first week of the pandemic outbreak (Moroń and Biolik-Moroń, 2021). In our study, trait EI seems to be related to coping strategies aligned with personal empowerment at the cognitive and behavioral levels in the context of the pandemic. Cognitively, high trait EI scorers were likely to reformulate positively the cognitive appraisal of the pandemic, without engaging in self-criticism and at the same time were able to proactively face its related hardships while keeping engaged, which is in opposition to learned helplessness.

Regarding the mediating roles of the various coping strategies, trait EI was related to psychological distress through maladaptive coping, and that was true for depression, anxiety, and stress (H2). Since we could not classify any of the coping strategies as adaptive following our data-driven criteria (see the Variable Selection and Assessment of the Measurement Model section), the mediating role of adaptive coping (H3a) could not be explored. Meaning-centered coping, on the other hand, partially mediated the relationship between trait EI and wellbeing. This provides partial support for the third hypothesis since trait EI predicted wellbeing directly but also indirectly through the adoption of meaning-centered coping.

Many studies have examined the factors that exacerbate the psychological effects of the pandemic (Figueroa and Aguilera, 2020; Sood, 2020), but there are fewer studies on the factors that might positively predict wellbeing or negatively predict the harmful psychological effects of the pandemic. The results of the present study highlight trait EI as a negative predictor of psychological distress within the context of the pandemic, namely, through coping strategies. This is consistent with the previously mentioned Lebanese study by Sanchez-Ruiz et al. (forthcoming) who found that trait EI negatively predicted substance abuse and denial and positively predicted acceptance, active coping, and positive coping, which were negatively related to anxiety and depression. This finding is also supported by the literature, whereby individuals with high trait EI were more likely to engage in adaptive rather than maladaptive (e.g., self-blame) coping strategies when confronted with stressful situations, which is related to less distress and greater wellbeing (Keefer et al., 2018).

In particular, emotion regulation, a core component of trait EI, allows individuals to choose coping strategies that downregulate their negative emotions while maintaining their positive emotions, which can be associated with less vulnerability to psychological distress and more resilience to stressful situations (Mikolajczak et al., 2008; Keefer et al., 2018). This resilience, rooted in the ability to choose adaptive coping strategies in the face of stressors and have a positive sense of internal control, is opposed to the maladaptive coping strategy of behavioral disengagement, which reflects the avoidance of stressors and a sense of uncontrollability over the subjective impact of stressful events (e.g., Varni et al., 2012). In the context of the pandemic, emotion regulation can possibly be the process through which individuals can maintain wellbeing.

A key novel contribution of the present study is the positive relationship found between trait EI and meaning-centered coping during the pandemic, which for the first time positions the former within the existential positive psychology framework. In accordance with this framework, our study showed that high trait EI scorers engaged in coping strategies that gave them a sense of meaning, and that predicted greater happiness with their lives and potentially fewer feelings of threat in the face of the pandemic. This emphasizes the idea that finding meaning through suffering is related to coping with stressful situations such as the pandemic. Indeed, the centrality of meaning-centered coping in mental health during the pandemic has been demonstrated in a recent study by Eisenbeck et al. (unpublished data) in 30 countries.

Consistent with the ideas of Frankl (1969, 1984) and Wong (1993, 2020), this existential way of coping, transforming adversity into growth through meaning and purposeful actions, plays a central role in dealing with the current pandemic. Since the relationship between trait EI and wellbeing can be explained by the proclivity of high trait EI individuals to choose and implement certain coping strategies over others (Mikolajczak et al., 2008), trait EI can provide the groundwork for individuals to be able to purposefully choose meaning-centered coping strategies, such as appreciating life and engaging in meaningful prosocial activities in the face of the challenges associated with the pandemic. In addition, trait EI and spiritual intelligence can be understood as constructs that have an interdependent relationship, whereby high trait EI can provide a strong foundation for strong spiritual intelligence, which in turn reinforces trait EI. This is because emotional competency may provide the foundation upon which existential–spiritual intelligence can be built on and developed, including, but not limited to, discovering personal meaning in life and improving transcendental awareness, which in turn, can improve wellbeing. Although the relationship between meaning in life has been explored with ability EI (e.g., Donohoe and Greene, 2009; Mohanty et al., 2015; Teques et al., 2016), there is a need to explore these relationships adopting the trait EI conceptualization, as in the case of the present study. Scientific research on the association between these two constructs is budding (Chin et al., 2012; Arbabisarjou et al., 2016) but needs further investigation. At the same time, these findings support the conceptualization of wellbeing by Wong and Bowers (2018), according to which sustained, attunement-based happiness during adversity requires significant levels of personal maturity, and this can be facilitated by the various components of trait EI, including emotion regulation, empathy, and optimism, among others.

Finally, finding meaning has been associated with better adjustment following collective traumas (Updegraff et al., 2008). Hence, these results are especially relevant for the Lebanese population, who is undergoing the same pandemic-related challenges as other countries worldwide, in addition to unprecedented and worsening financial, social, and political instability. These circumstances can set the grounds for an existential crisis with pervasive effects in terms of anxiety and stress in the face of unknown repercussions and a bleak and uncertain future.

### Practical Implications

Overall, the current study is one of the very few in the literature indicating that the understanding and management of one’s own and others’ emotions, which are core elements of trait EI, can be linked to the restoration and sustainment of meaning in life and wellbeing, especially in times of adversity—namely, the COVID-19 pandemic. A series of recent studies have emphasized the need for developing appropriate interventions for individuals impacted by the pandemic (Duan and Zhu, 2020; Wang et al., 2020), and some have highlighted the protective role of EI, using both trait (Moroń and Biolik-Moroń, 2021) and ability measures (Prentice et al., 2020), against the negative psychological consequences of the pandemic. Sood (2020) and Figueroa and Aguilera (2020) also stressed on the importance of using technological tools, such as virtual counseling or support groups, which would better equip the population for the lasting effects of this pandemic. As meaning-centered coping was found to partially mediate the relationship between trait EI and wellbeing, the present study provides support for incorporating principles that fall under meaning-centered coping, such as appreciating life and engaging in prosocial activities, into interventions useful in this pandemic and other stressful situations, consistent with aforementioned studies.

Our study also emphasizes the importance of interventions that focus on increasing trait EI, since the results show that trait EI contributes in a unique way to the development of wellbeing in addition to its effect through meaning-centered coping. For example, trainings designed to target emotional competencies have shown to lead to improvements in wellbeing, quality of life, and social interactions, among others (e.g., Nelis et al., 2011). The present study adds to the increasing body of evidence about the benefits of trait EI and its implications, and is one of the first in pointing out its key role during the pandemic. Our findings can inform psychological interventions, which can benefit from incorporating both trait EI and meaning-centered coping, especially for individuals in stressful situations and those exposed to trauma. Indeed, research on meaning-centered therapies has found that they have lasting positive effects, particularly with regard to maintained hope and optimism, and are effective in helping individuals cope effectively with stress (Vos, 2016). In addition, recent studies have emphasized the importance of these interventions during the pandemic (e.g., Gaj and Castiglioni, 2020). Schnell and Krampe (2020) also concluded that it is necessary to face existential concerns and focus on one’s goals in order to find meaning that can help individuals cope with the ongoing crises and minimize distress. Taken together, the results support the utility of the existential positive psychology framework to curb the psychologically distressing effects of the pandemic.

### Limitations and Recommendations for Future Studies

This study attempted to overcome the limitations associated with the different conceptualizations of the coping strategies by following a data-driven approach in categorizing maladaptive and adaptive strategies. However, there are potential drawbacks of the present research, such as the cross-sectional and correlational design, which does not allow inferring causality. Another limitation is the measurement method, which relied entirely on self-reported data, thus allowing the possibility of the mono-method bias. Additionally, even though the internal consistencies in this sample were adequate, some of the measures used have not been previously validated in Lebanon. As an example, the DASS-21 has not been previously validated in Lebanon; however, such validation is ongoing (Nassar et al., forthcoming), and the measure has been administered in both Lebanese and Middle Eastern samples showing satisfactory psychometric properties (e.g., Bener et al., 2016; Hamaideh, 2017; Fawaz and Samaha, 2020).

Future studies could target larger and more diverse samples and incorporate some objective measures to investigate coping mechanisms during the pandemic. Longitudinal research is recommended in order to elucidate the long-term influences across the different waves of the pandemic. In addition, the role of meaning in life as a coping mechanism can be explored under different life stressors. Specifically in Lebanon, it would be valuable for upcoming research to explore the differential role of meaning in life for those who have experienced trauma (civil war, or more recently, the Beirut explosion of August 4) and those suffering from the severe sociopolitical crises the country is currently undergoing. Finally, conducting intervention studies via randomized control trials is also needed to further investigate the preventative and healing effects of meaning-centered coping strategies rooted in existential positive psychology. Such studies could also incorporate more complex modeling to account for various situational and psychosocial variables (e.g., social support) that may be at play.

## Data Availability Statement

The datasets generated for this study are available upon request to the corresponding author.

## Ethics Statement

The studies involving human participants were reviewed and approved by Institutional Review Board Lebanese American University. The patients/participants provided their written informed consent to participate in this study.

## Author Contributions

M-J S-R is the principal investigator. She is the primary person responsible for the conceptualization and crystallization of the main idea of this article. She coordinated and participated in the writing-up of the manuscript. NT and TK contributed to the conceptualization of the study. They collected the data and contributed to the writing of the original draft, editing, and review of the manuscript. VE contributed to the literature review by gathering the necessary references, assisted in the writing-up of the manuscript, and presented the figure describing the mediational model. NE and DC constructed the survey and assisted in positioning this specific article within the existential positive psychology framework through their valuable comments, suggestions, and review. They also contributed to the design of the SEM model. EN contributed to the construction of the SEM model and conducted the statistical analyses reported within this article. She drafted the methodology and Results section, including the tables. As the corresponding author, she also handled the submission process and ensured the compliance to author guidelines. All authors contributed to the article and approved the submitted version.

## Conflict of Interest

The authors declare that the research was conducted in the absence of any commercial or financial relationships that could be construed as a potential conflict of interest.
